# End-to-End QoS “Smart Queue” Management Algorithms and Traffic Prioritization Mechanisms for Narrow-Band Internet of Things Services in 4G/5G Networks

**DOI:** 10.3390/s20082324

**Published:** 2020-04-19

**Authors:** Mykola Beshley, Natalia Kryvinska, Marian Seliuchenko, Halyna Beshley, Elhadi M. Shakshuki, Ansar-Ul-Haque Yasar

**Affiliations:** 1 Department of telecommunications, Lviv Polytechnic National University, Bandery 12, 79013 Lviv, Ukraine; mykola.i.beshlei@lpnu.ua (M.B.); m.seliuchenko@gmail.com (M.S.); halink@ukr.net (H.B.); 2 Department of e-Business, School of Business, Economics and Statistics, University of Vienna, A-1090 Vienna, Austria; 3 Department of Information Systems, Faculty of Management, Comenius University in Bratislava, 82005 Bratislava 25, Slovakia; 4 Jodrey School of Computer Science, Acadia University, 27 University Ave, Wolfville, NS B4P 2P7, Canada; elhadi.shakshuki@acadiau.ca; 5 Transportation Research Institute, Hasselt University, BE3500 Hasselt, Belgium; ansar.yasar@uhasselt.be

**Keywords:** Internet of Things (IoT), Long-Term Evolution (LTE) standard for wireless broadband, Narrow-Band IoT (NB-IoT), prioritization, Quality of Services (QoS), traffic scheduling, 4G/5G broadband cellular network technology

## Abstract

This paper proposes a modified architecture of the Long-Term Evolution (LTE) mobile network to provide services for the Internet of Things (IoT). This is achieved by allocating a narrow bandwidth and transferring the scheduling functions from the eNodeB base station to an NB-IoT controller. A method for allocating uplink and downlink resources of the LTE/NB-IoT hybrid technology is applied to ensure the Quality of Service (QoS) from end-to-end. This method considers scheduling traffic/resources on the NB-IoT controller, which allows eNodeB planning to remain unchanged. This paper also proposes a prioritization approach within the IoT traffic to provide End-to-End (E2E) QoS in the integrated LTE/NB-IoT network. Further, we develop “smart queue” management algorithms for the IoT traffic prioritization. To demonstrate the feasibility of our approach, we performed a number of experiments using simulations. We concluded that our proposed approach ensures high end-to-end QoS of the real-time traffic by reducing the average end-to-end transmission delay.

## 1. Introduction

Internet of Things along with Big Data, virtualization and fifth-generation mobile networks (5G) make one of the most promising areas of today’s development technologies [1]. IoT provides more than 50 applications’ areas; to name a few, this includes intelligent sensors (for electricity, gas, water), facility management, home and commercial real-estate security and fire alarm systems, personal electronic health sensors, human, animal or object tracking systems, smart city infrastructure (e.g., street lamps or trash canisters, smart home and connected industrial tools).

With developments of the Internet of Things, the number of connections to mobile networks is increasing steadily. According to the Ericsson forecast [2], by 2021, the total number of devices connected to the internet in the world will be around 28 billion. In the years to come, the number of the intermachine connections, i.e., Machine-to-Machine (M2M) will grow by 25% per year. The majority of M2M devices delivered to the market will support the LTE standard. As the IoT market grows, it is clear that existing mobile technologies are insufficient for many of these solutions due to insufficient coverage, high terminal costs and short battery life.

An innovative IoT technology solution is the Narrowband IoT (NB-IoT) standard. This is a wireless narrowband type of global networks with a Low Power Wide Area Network (LPWAN) radio technology standard developed by 3GPP to enable a wide range of cellular devices and services, which is primarily designed for machine-to-machine applications [3,4,5]. NB-IoT technology occupies a low-speed niche in the class of solutions, where the priority is uninterrupted data transmission and low power consumption.

Further to this, as new technologies are developed such as narrowband LoRaWAN, Long Range Wide Area Network, and NB-IoT, it is expected in the longer run 5G telecom operators will occupy new niches, offering not only the communication service but also a comprehensive solution, including system integration services and IoT service platforms [3,4]. This trend has already emerged. In December 2017, it was agreed that 3GPP standards for new radio networks (NR) will be capable of operating together with the existing Long-Term Evolution (LTE) standard for wireless broadband networks that use a non-standalone (NSA) mode for mobile super-broadband communications. In June 2018, 3GPP standards’ Release 15 for 5G NR standalone (SA) mode was completed [6]. The evolution of 5G standards described in the specifications of Release 16 of 3GPP is planned for use in early 2020 [7], with more enhancements set to follow in Release 17 [8].

Besides the main narrowband, the integration to the mobile network is possible for functions on the LTE standards (e.g., LoRaWan, Strizh, Sigfox, NB CloT, NB-LTE, Wireless RF, Bluetooth, Wi-Fi, and Zigbee). All of these technologies have their advantages and disadvantages [9]. However, there is one common problem that concerns both static and dynamic devices such as sensors, which involves the use of a spectrum that can be either licensed or unlicensed. The license spectrum provides more opportunities for quality of service than the unlicensed spectrum. When transferring data to the unlicensed spectrum, there is virtually no guarantee that the data will be delivered to its destination in a timely manner.

The benefits of implementing IoT based on LTE architecture are as follows:

1. The QoS is guaranteed when using the license spectrum.
2. It has a response delay time less than 10 ms.
3. The flexibility of managing the QoS.
4. The allocation of network resources is adaptive.
5. The level of system security is high.
6. With IPv6 addressing support, it provides high scalability.
7. The network architecture provides the basis for the implementation of new services and providing Device-to-Device interaction.
8. It is able to serve the growing number of devices with high transmission rates.
9. It offers roaming support [10,11].

In 4G/5G networks, it should use a “smart queue”, where priority depends on the type of service [12,13,14,15]. Different types of services require different prioritization. For example, VoIP video conferencing requires low jitter and latency, while other applications (e.g., remote surgery) require a guaranteed level of reliability, while multimedia requires guaranteed channel bandwidth. Within the QoS requirements, all types of services are divided into nine classes, each of which corresponds to a QCI (QoS Class Identifier) [16]. In addition, the end-to-end channels organized for traffic transmission are divided into two groups in accordance with the type of resource allocated:

- with guaranteed GBR (Guaranteed Bit Rate);
- with an unguaranteed Non-GBR (Non-Guaranteed Bit Rate) transmission rate.

The GBR transmission service is used for applications when providing real-time services. Each GBR transmission service channel is associated with a given value of the QoS parameter. If the traffic transmitted by the GBR service corresponds to the value associated with the GBR service, then there are no problems associated with overload during transmission of this data and packet loss. The non-GBR service does not guarantee any specific data transmission speeds for services on the LTE network. This service is mainly used for applications such as web browsing and FTP transmission. A non-GBR channel service is highly susceptible to packet loss due to network congestion and also does not block any specific transmission resources in the LTE network.

At present, the LTE network provides the required QoS for various IoT services, for example LTE-M. The main method for ensuring QoS is the use of communication channels with high bandwidth, for example in [17], the authors proposed MTC (Machine type communication) QCIs for GBR and non-GBR resource types to prioritize IoT traffic in LTE-based networks. However, this is an expensive method. The essence of other methods is to give priority to the provision of network traffic resources due to protocols that do not require high quality of service [18,19]. NB-IoT is considered as a basic communication standard for the development of the IoT applications. When it is compared to competing solutions, it is characterized by its energy efficiency and the ability to transmit small messages at high speed. With NB-IoT, operators are not required to rebuild their infrastructure; however, updating the software on the base station is sufficient to ensure the necessary coverage [20]. NB-IoT technology is now implemented in the standards of the 3rd Generation Partnership Project (3GPP), where low-bandwidth IoT devices can provide secure access to the fifth generation (5G) core network via the 3GPP access network [21]. The use of a narrow bandwidth of 180 kHz in NB-IoT has following limitations to effectively manage the handover; the quality of services; to guarantee the data transmission speed and total E2E delay, which significantly reduced the possibility of Internet services of things in NB-IoT networks and limited the use of NB-IoT for Industrial Internet; Tactile Internet; unmanned transport; medical robots. However, NB-IoT technology does not differentiate between individual streams from IoT devices, taking into account their QoS requirements [22]. The end results are suboptimal load balancing and deterioration of the quality of service for real-time traffic, because the GBR (Guaranteed Bit Rate) bearer is not created for NB-IoT RAT type [23,24,25].

With the development of IoT services, it is natural that one of the main tasks is to adapt QoS according to the requirements of a particular type of service. Thus, the mechanisms of the traffic prioritization in 4G/5G networks for the NB-IoT systems/services is one of the most important aspects on which the development of the IoTs will depend in the future [26,27,28].

When a maintenance is performed at 4G/5G base stations deployed on the basis of LTE technology, a certain memory buffer is dedicated to serve as a queue. When packages are transferred, they are replaced by new ones. In order to eliminate failures, additional buffer is reserved to serve as a certain extension to the queue. This will reduce the queues or waiting time for a service. Since most of the NB-IoT data is transmitted over an uplink, the random access channel (i.e., the main interface between the devices and the base station) can usually become a major bottleneck for the entire system. To improve QoS parameters, an effective RACH (Random Access Channel) procedure is required to increase the success of the RACH, especially with regard to the interaction between the static properties of the physical radio channels and the dynamic properties of the queue developing in each IoT device. The authors of work [29] in detail present the implementation of an NB-IoT random access modeling tool with open source code and in accordance with 3GPP, as well as an analytical model describing both the collision probability, the probability of success and end-to-end delay given the number of users accessing the random access channel. 

When using allocated resources, it is important to know what data rate is set for packets’ transmission. Further, speed allocation is one the necessary requirements due to the fact that some packets may require a guaranteed speed, while others do not. The other requirement is distance, especially for the delivery speed of a request to a device needed to perform certain actions. After all, the request traverses from its source to its destination visiting several intermediate nodes and creates an additional delay. It is also necessary to take into account the fact that in this case, intermediate memory buffers are required, because the loss of packets is possible anywhere along the transmission link [30,31]. It is also important to consider the mobility of users who want to access the device under their supervision. This means that the service is dynamic and moves from one station to another; thus, the range of the base station is changing. This impacts the packet transmission speed. This means that the further the subscriber is from the base station, the lower the transmission speed. Therefore, this situation affects the speed of the device to perform the desired actions [32]. In general, improving the quality of service requires monitoring the network. At the same time, it is possible to record experienced bottlenecks and as such, new methods are explored to eliminate them. Therefore, QoS level increase is an important stage of network development. It consists of modernization and installation of new elements [33]. These are necessary for the qualitative provision of services in 4G/5G networks for IoT services [34,35,36,37,38].

We found that NB-IoT technology is a promising technology for providing IoT services. The technology only supports non-guaranteed delivery services (non-GBR). The non-GBR service does not guarantee any specific data transmission speeds for services on the NB-IoT network. This service is mainly used for unreal time traffic, for example, consumer IoT applications. A non-GBR channel service is highly susceptible to packet loss due to network congestion and also does not block any specific transmission resources in the NB-IoT network. 

Therefore, the research problem is that NB-IoT has some limitations, one of them being that a GBR bearer is not created for NB-IoT RAT type and cannot guarantee end-to-end QoS requirements (E2E delay) for real time IoT traffic. That is why in our work for the future development of NB-IoT technology, we propose the idea of providing GBR to ensure ultra-low delay of IoT traffic. Thus, in addition to its existing advantages, NB-IoT will be suitable for providing services with critical levels of requirements for priority, reliability and delay for Industrial Internet of Things services, which is important for LTE-based existing 4G and future LTE-based 5G networks.

The purpose of our work is to ensure the guaranteed QoS for NB-IoT services. This is achieved by developing methods of service quality management from end-to-end; namely, methods of IoT traffic prioritization (to realize GBR and non-GBR classes for NB-IoT), channel formation, and distribution of its resources in 4G/5G networks deployed on the basis of LTE technology.

The rest of the paper is organized as follows. Section 2 describes the related research work on the development of the management algorithms and traffic prioritization mechanisms for IoT services in LTE-based 4G/5G networks. We start with the development of an improved architecture of the LTE mobile network. This is to provide IoT services by allocating a narrow bandwidth and transferring controlling/signaling functions from the eNodeB base station to the NB-IoT controller (Section 3). Further to this, we develop smart queue management algorithms based on the 4G/5G IoT traffic prioritization method (Section 4). Then, we apply a method for prioritizing the IoT traffic to ensure E2E QoS in heterogeneous LTE /IoT networks, with a distribution of the uplink and downlink resources of NB-IoT technology (Section 5). Next, we prove the effectiveness of the proposed solutions using a simulation model of a heterogeneous mobile network LTE/IoT, which allows testing scenarios of IoT devices’ behavior and a service of subscribers close to the real ones by providing QoS (Section 6). Finally, Section 7 concludes this work.

## 2. Related Work

In this part, we illustrate the research status of development of the management algorithms and traffic prioritization mechanisms for IoT services in LTE-based 4G/5G networks. Nowadays, only one percent of the things that are around us are connected to the internet. This constitutes the current IoT which equals to around 50 billion devices, including sensors and mobile users. It is expected that this number will increase to one trillion devices by the year 2022 [39]. Therefore, a mobile network technology is required that is able to handle this number of connected devices to the internet. In the first stages, the new technology is developed on the basis of 4G (LTE) infrastructure. For the future development of IoTs, the 5G LTE infrastructure will be complemented by innovative radio access developments, new approaches and quality management techniques, and traffic streaming mechanisms. At the physical layer, multi-standard antennas will be used with a large number of antenna elements to support a wide spectrum of frequencies, along with software solutions that improve the coordination of the work at the base stations [40]. 

In this research paper [41], the authors conduct a comprehensive study of NB-IoT and LoRa as effective solutions for connecting devices. It showed that unlicensed LoRa technology has advantages in terms of lifetime, capacity and battery cost. At the same time, licensed NB-IoT has advantages in terms of QoS, latency, reliability and range.

The research discussed in [42] provides an overview of NB-IoT design based on LTE infrastructure, which includes salient features from the physical and higher layers. The work presented in [43] considers three deployment modes supported by NB-IoT, including standalone operation, guard-band operation, and in-band operation. The paper discussed in [44] describes that NB-IoT is the newest technology of mobile radio access based on Long-Term Evolution (LTE) implemented in the framework of the Third-Generation Partnership Project (3GPP). It is shown that NB-IoT is based on LTE design with some modifications to meet IOT requirements. For example, at the physical (PHY) level, only modules with a single antenna and low order are supported, while at the Medium Access Control level (MAC), only one physical block of resources is allocated for resource planning. The paper also includes an overview of Evolved Packet Core (EPC) changes to support the Service Capability Exposure Function (SCEF) to manage both IP and non-IP data packets over the Control Plane (CP) and User Plane (UP), possible NB-IoT deployment scenarios in future heterogeneous wireless networks (HetNet). This work provides a comprehensive overview of the NB-IoT standard from Release 13 to Release 16, with the aim of improving and ensuring more realistic research.

In [45], the authors consider the impact of the protocol stack on the performance of the narrowband channel NB-IoT. The authors found that while CoAP/UDP (Constrained Application Protocol/User Datagram Protocol-based transport stably works better in terms of both latency, coverage, and system bandwidth, MQTT/TCP (Message Queuing Telemetry Transport/Transmission Control Protocol) also works when the system is less busy.

The paper [46] focuses on the construction of the NB-IoT simulation model based on OPNET and testing its characteristics, such as wide coverage and high channel load. The authors mainly consider the design and implementation of existing NB-IoT technology in terms of characteristics of the physical layer of NB-IoT based on a Long-Term Evolution (LTE) network. The process of NB-IoT functioning is studied when integrating with LTE networks with 3 MHz, 5 MHz, 10 MHz, 15 MHz and 20 MHz channels capacity. The simulation results confirmed the performance of NB-IoT, where the uplink delay is less than 10 s, the channel utilization is higher than in the LTE network, and the coverage area is larger than in the LTE network. It is also shown that NB-IoT will be used for non-delay-critical internet applications due to quality of service limitations. If you need to implement NB-IoT based on LTE access networks for critical services, you need to improve NB-IoT technology.

The authors [47] systematically measure the physical layer as well as verify application layer performance. Particular attention is paid to the impact of radio parameters on the application layer QoS. The work investigates non real-time services due to the fact that the existing NB-IoT technology is not suitable for critical services that require low latency, and requires improvement in the technology NB-IoT.

In [48], Cheng and his colleagues solve the problem of congestion in the NB-IoT network, which occurs as a result of accidental access by multiple devices. For this purpose, the authors have proposed an algorithm for optimizing random access and created random access with differentiated barring (RADB), which may increase the insufficiency of the traditional method of dynamic access bandwidth class. Finally, the algorithms proposed in this paper are implemented with the NB-IoT model installed using the OPNET Modeler platform, and modeling is performed. The results of the modeling show that RADB is able to effectively solve the conflicts of the requests arising from random access to multiple devices, and mainly provide effective and reliable random access for devices sensitive to delays.

The authors in [49] have described two uplink physical channels defined by NB-IoT, including NPUSCH and NPRACH. An event-aware back pressure scheduling scheme for emergency IoT, which is to polish up the NB-IoT system QoS, has been proposed by Liu, J. et al. [50]. In [51], Zhang and his colleagues described the QoS provisioning for IoT in LTE-A Heterogeneous Networks (HetNets) with Partial Spectrum Usage (PSU). In HetNets, IoT users with ubiquitous mobility support or low-rate services requirements can connect with MacroCells (MCells), while FemtoCells (FCells) with PSU mechanism can be deployed to serve the IoT users requiring high-data-rate transmissions within small coverage.

Recently, Chen et al. in [52] proposed service quality management solutions for IoT. Chen with his co-workers put forward an improved K-means algorithm to cluster the NB-IoT devices. They assign an initial priority for the cluster. Then, they proposed a priority generation algorithm for the tasks in the buffered queue. According to the integral priority of the tasks, the base station scheduler allocates services for the waiting tasks in the queue. The disadvantage of such a solution is the complexity of the implementation on the real network, as it requires a complete update of the software resource planner at the base station. It is not clear how this solution will affect the operation of the entire 5G mobile network. It should be noted that the resource plans of the base station are also responsible for allocating resources to mobile users where their own algorithms of priority service exist [53].

## 3. Launching NB-IoT Controller for Management of IoT Traffic with E2E QoS over LTE-Based 4G/5G Networks 

Recently, the active development of network technologies has led to the emergence of new applications, such as online games, distance learning, and robots’ management. The flow generated by these applications is sensitive to delays. However, compared to VoIP or video, they impose stricter restriction on the time of delivery, which is about 10 ms. For some applications (e.g., remote surgical operations), it is necessary to provide shorter delivery time to a maximum of 1 ms. A concept of a network interaction, involving a transfer of data with ultra-short delays, is called the Tactile IoT [54,55].

The ability to meet the specified requirements for the quality of service in LTE networks largely depends on how the base station is planning the transmission of various packets, using the resources of the wireless channel. The scheduler at the base station is responsible for planning radio resources. There are many planners/schedulers available on the market [56]. They provide services for both streams that are sensitive and insensitive. Our concern is that if it is possible to utilize the existing schedulers for transferring data from a Tactile IoT. In LTE and NB-IoT technologies, the downlink and uplink channel planning mechanisms are not defined by the standard, leaving the choice for the manufacturers of base station equipment eNodeB [57]. 

**Figure 1:**
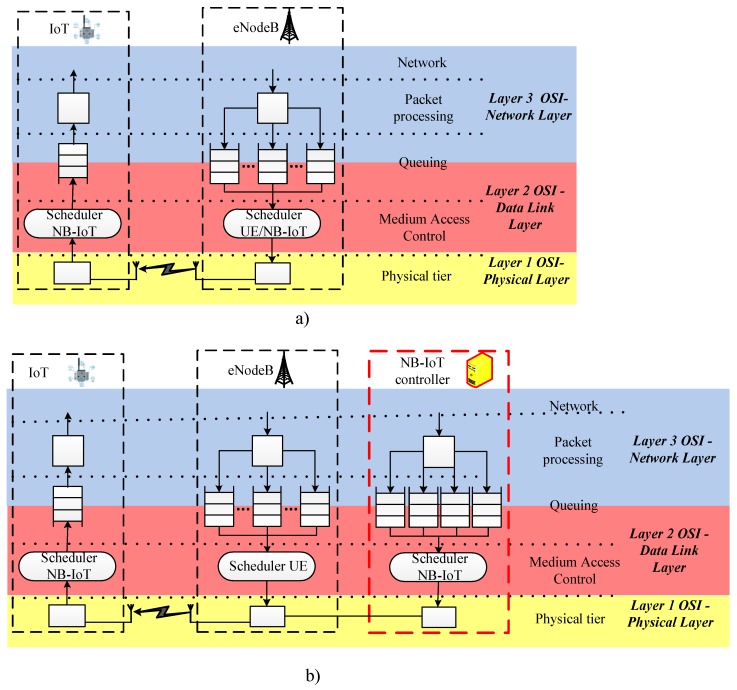
Classical Narrow-Band Internet of Things (NB-IoT) Architecture of 4G/5G base station interworking with IoT device (**a**), and the proposed NB-IoT architecture of interactions between 4G/5G base station, IoT controller and IoT device (**b**).

The significant increase in the number of IoT devices led to certain problems in modern mobile networks. Despite the fact that the capacity of 4G/5G networks is sufficient to meet the needs of most devices, the signal load generated by them exceeds the capabilities of the base stations. To solve this problem, we utilize the classical architecture of LTE-based 4G/5G base station interworking with the NB-IoT device, which is shown in Figure 1a. To minimize the changes in the LTE network and the corresponding equipment costs, it is suggested that an NB-IoT controller be introduced into the architecture [58]. Our proposed functional structure and interactions between a base station, an NB-IoT controller, and an IoT device is shown in Figure 1b.

This structure consisting of four tiers that are listed below:

1. Packet processing tier.
2. Queue tier for data transmission over a wireless communication channel.
3. Medium access control.
4. Physical tier [44,56].

This controller will be responsible for the mechanisms of downlink and uplink channel planning for IoT devices and will allow network operators to leave existing base stations eNodeB unchanged. The controller is a separate server machine on which the software responsible for IoT traffic resources planning (scheduler) is installed. It is possible to install this controller near the LTE base station or deployed in the cloud with the possibility of renting and increasing the performance of the server. The implementation does depend on the predicted number of IoT devices connected to the base station. Significant growth in the number of connected devices requires a powerful server machine for fast operation of the IoT controller. However, to enable IoT services, network designers need to separately allocate a narrowband spectrum of 200 KHz. There is no need for high bandwidth due to transmission of small volumes of data. If it is necessary to provide high speeds, it is proposed to transmit data in the spectrum of LTE.

We propose to define priority classes where each class includes traffic of a particular NB-IoT based on the QoS Class Identifier (QCI) parameter. The QCI parameter can take one of the nine states, each of which is associated with a certain Type-of-Service (ToS), and thus with the type of the transmission channel, rate, error rate, and delay. The QCI is a label in the IPv6 “Channel ID” package. 

For IoT services, we propose to use a prioritization method that is based on the criterion of allowable delays and the average number of service failures [59,60]. According to Table 1, there are four IoT services classes with different QoS requirements. QCI_IoT_ is a label in an IPv6 package and is a part of the ToS field.

In most cases, IoT devices will be assigned a certain priority in advance depending on their purpose, and all messages that will be sent will have a defined priority. Each priority has its own acceptable quality of service parameters that must be ensured. In particular, the main parameter is the delay, the acceptable values of which are shown in Table 1. When a low priority reaches its maximum delay in the proposed smart queue, the counter that monitors the waiting time of the priority message will decide to immediately allocate resources to it for transmission. After that, this message becomes the highest priority, but physically it does not change dynamically in marking by a QCI identifier. A detailed description of the smart queue management algorithms for IoT Services is described in the next Section 4. Unlike the standard known structure, our proposed structure reduces the overload from the base station when planning radio resources. It also provides the required QoS for IoT services, taking into account their priorities.

## 4. Development of “Smart Queue” Management Algorithms for IoT Services

This section describes our proposed “smart queue” management algorithms. Modern 4G/5G networks provide the required QoS for various services. To this end, we propose to use the “smart queue” concept, in which a priority depends on the type of service. Different types of services require different prioritization. As such, one of the main tasks with the development of IoT services is to adapt the QoS in accordance with the requirements of a specific type of service. Delivering services over mobile networks should consider not only the priority but also the delay, the requested speed of service execution, and a guaranteed performance. With regard to the latter, when it comes to the exchange of data between devices, it is important to agree on QoS parameters that must be ensured at both ends of the transmission. To enable priority, we suggest that certain memory buffers should be formed on the proposed IoT controller to serve as a queue. As the packets are sent, the locations of sent packets in the queue are released for the incoming packets. To avoid service denial, an additional buffer is installed. The additional buffer serves as an extension to the queue, and at the same time, decreases queuing delays for high priority packets.

In this work, we propose algorithms for managing “smart queue” based on the proposed prioritization of IoT traffic in heterogeneous mobile network.

### 4.1. Types of Real Time Traffic with Guaranteed Transmission Delay (GBR_IoT_)

This section provides a detailed description of our proposed IoT class L1, IoT class L2, IoT class L3, and IoT class L4, where L1 is the highest priority and L4 is the lowest priority. Our proposed algorithms are described by means of flowcharts. 

**Figure 2:**
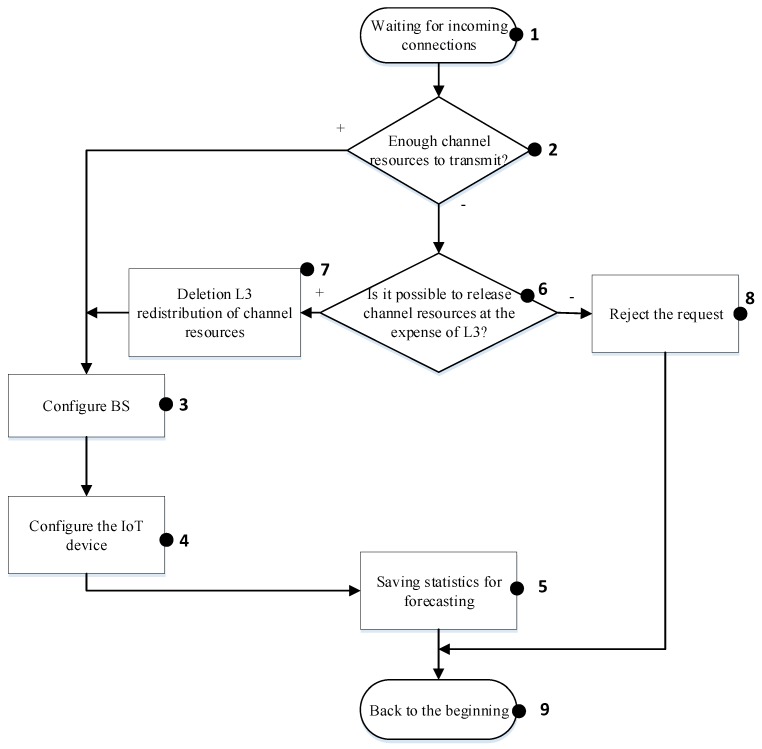
Algorithm for managing the “smart queue” for IoT class L1.

The purpose of the statistics collection module is to determine the traffic parameters, both general and for each specific protocol, registration of unknown traffic, or traffic analyzed with errors. A general algorithm of the analyzer’s program operation is depicted in Figure 2.

#### 4.1.1. IoT Class L1

Figure 2 shows the control flow of the proposed algorithm for IoT class 1. When the algorithm is initialized, it starts at the base station waiting for the data transfer request (block 1). In case a request is received, the base station analyzes the priority of the device from which the transmission took place. It determines that the priority of the device is L1 (the highest). Consequently, the base station analyzes the queue and the network resources that are available for data transfer (block 2). If there are available resources, then the base station reconfigures itself (block 3) and responds to the IoT device with the signaling data containing information about the allocated resources (block 4). When a device finishes the transmission, the base station saves statistical data (block 5) for future analyses and prediction of the IoT device activity, and then goes to block 1. Conversely, the possibility of releasing resources through the devices of class L3 (block 6) is checked. If there are no available resources for the IoT device with the highest priority, then the base station may allocate the required resources by reorganizing the queue and delaying transmission for lower priority (L3) IoT devices (block 7). Otherwise, the request is rejected (block 8) and the algorithm is terminated. 

#### 4.1.2. IoT Class L2

**Figure 3:**
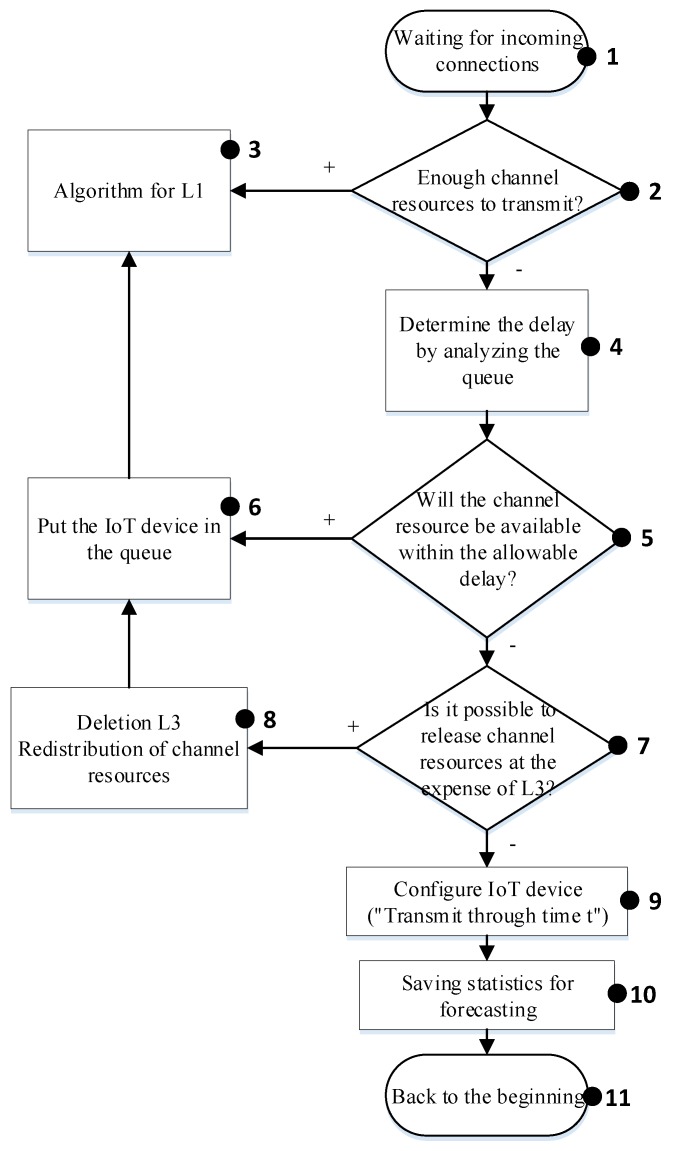
Algorithm for managing the “smart queue” for IoT class L2.

The control flow of the proposed algorithm for IoT class 2 is shown in Figure 3. At the beginning, the base station eNodeB is waiting for the connection setup request (block 1). During the processing of each request, the class of the requested device is determined by considering the type of traffic it wants to transmit. All processed requests are stored in the queue based on their priority. On the basis of the received data, the availability of resources for traffic transmission from the devices of class L2 (block 2) is checked. If the resources are sufficient, then the algorithm for L1 (block 3) is executed. On the other hand, if the resources are not sufficient, then the queue is analyzed, and the delay of the transmission is determined (block 4). The availability of the resources within the tolerable delay for a given class is checked (block 5). In the case of the availability of sufficient resources and the possibility of allocation within the allowable delay, then the request from IoT device is queued and the algorithm proceeds to L1 (block 6 → 3). In the opposite case, the possibility of releasing resources allocated for the devices of class L3 (block 7) is checked. If it is possible to release the resources, then the IoT class L3 device is delayed and the queue is reordered (block 8). Otherwise, the IoT device is configured for transmission (block 9). The statistical data are stored for future analyses and prediction (block 10). In the end, this process will repeat again. 

### 4.2. Types of Non-Real Time Traffic with Non-Guaranteed Transmission Delay (Non-GBR_IoT_)

#### 4.2.1. IoT Class L3

**Figure 4:**
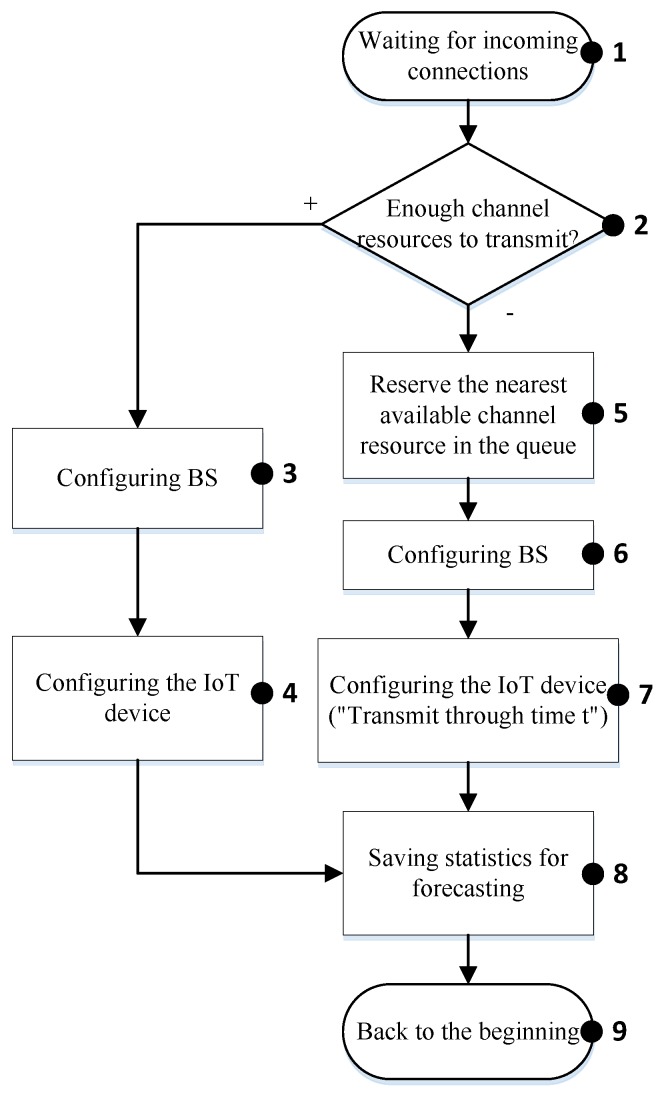
Block-diagram of the algorithm for managing “smart queue” for IoT class L3.

The proposed algorithm for IoT class 3 is shown in Figure 4. At the start of the algorithm, the base station (BS) begins to wait for the data transfer request (block 1). It is determined that the device priority is L3. An analysis of the queue and network resources for data transfer (block 2) is executed. If there are available resources, then the corresponding configuration is applied to the base station (block 3). Meanwhile, the required data and the transmission approval are sent to the IoT device (block 4). After a successful transfer, the statistical data are saved (block 8) for future analyses and prediction of the activity of the IoT device. 

If the available resources are insufficient, then the queue analysis is carried out and the resource is allocated immediately (block 5). The base station is configured for data transmission (block 6) and the starting time of the transmission *t* is calculated. The signaling data, the delay time *t* and the transmission request approval are sent to the IoT device (block 7). After a successful transfer, the statistical data are saved (block 8). At this point the algorithm ends (block 9). 

#### 4.2.2. IoT Class L4

**Figure 5:**
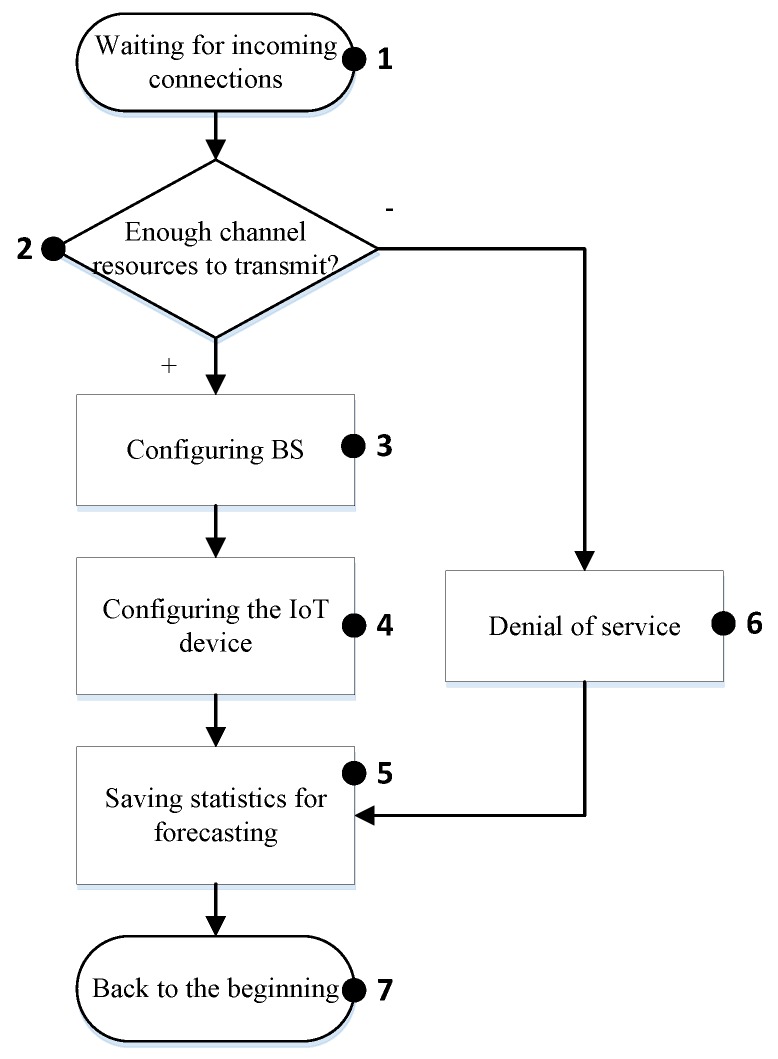
Block-diagram of the algorithm for managing “smart queue” for IoT classes L4.

The proposed algorithm for IoT class 4 is shown in Figure 5. At the beginning of the algorithm, the base station starts waiting for the data transfer request (block 1). It is determined that the device priority is L4. An analysis of the queue and network resources for data transfer (block 2) is executed. If the available resources are found, then the proper base station configuration is applied (block 3), and the signaling data and the transmission request approval are sent to the IoT device (block 4). After the successful transfer, the statistical data is stored (block 5) for future analyses and prediction for the activity of the IoT device. If there are not enough available resources, then the data transfer does not occur. Accordingly, the device receives a denial of service (block 6). There is a record of unsuccessful transmission (block 5) and thus the algorithm ends.

**Figure 6:**
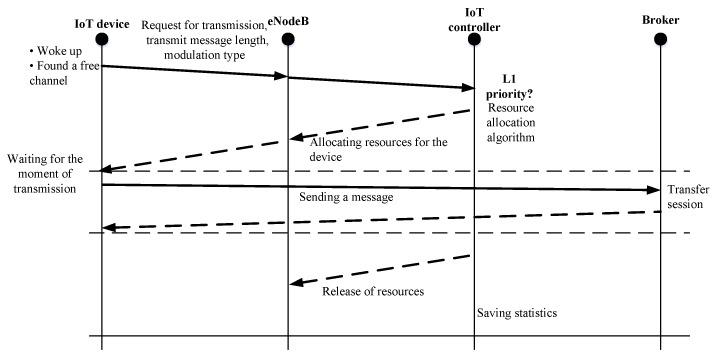
Interaction of Long-Term Evolution/Internet of Things LTE/IoT network elements when transmitting L1 message.

Figure 6 shows the sequence diagram to demonstrate the interaction between LTE/IoT network elements (IoT device, eNodeB, IoT controller, and Broker), while transmitting the L1 message. When the IoT device “woke up” reads the sensor information, it creates a message and sends a transmission request to eNodeB. This request contains the message size and modulation type based on the signal level and is forwarded to IoT controller. The IoT controller then analyzes the request and allocates the required resources at the base station for transmission. When the IoT device receives the response, it waits for its transmission time and then transmits the message. After transmitting the IoT message, the controller releases the resources at the base station and saves all generated statistical data. A broker is a server that receives information from publishers and transmits it to the appropriate subscribers. In complex systems, the broker can also perform various operations related to the analysis and processing of incoming data. The broker can set priorities for messages and create queues for message transmission. Thus, the broker organizes the sending of messages, their storage and filtering. Message queue is a container or a block where messages are stored while they are being sent. If the communication channel resource is insufficient or if the recipient is unavailable while sending the message, the queue stores the message until it is delivered.

In the next section, we discuss our development of downlink/uplink modified resources.

## 5. Services Resources Distribution in the IoT Downlink and Uplink Channels Using Proposed Smart Queue Concept

**Figure 7:**
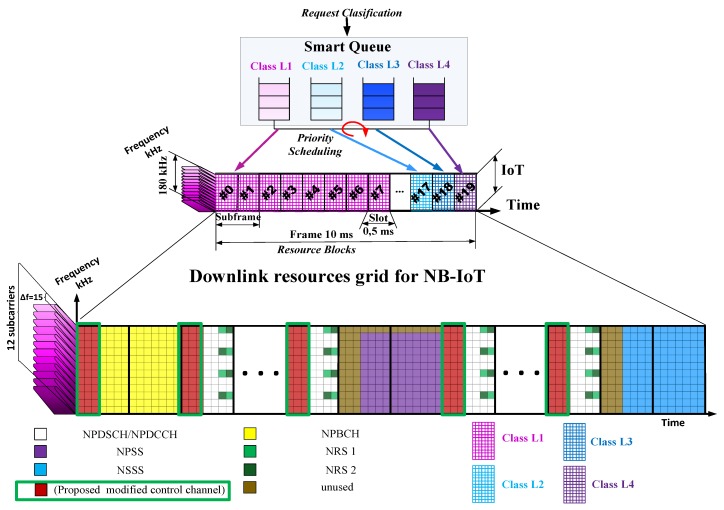
Resource grid of LTE/IoT for the downlink channel.

**Figure 8:**
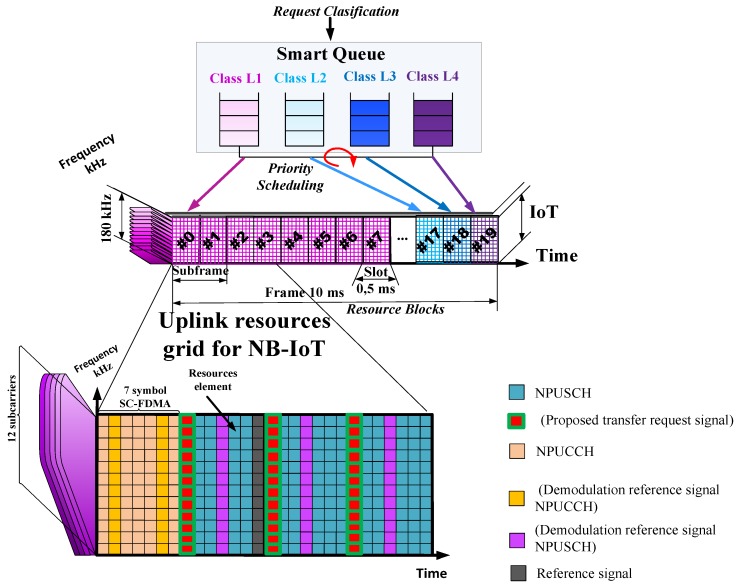
Resource grid of LTE/IoT for the uplink channel.

The most effective result in providing the required level of service quality in LTE is achieved by tackling the problem of frequency and time allocation in the downlink and uplink channels. Thus, we propose to allocate and distribute the resources in the downlink and uplink of IoT, using our proposed smart queue approach. Within the context of the narrowband IoT in the LTE mobile network introduction, the downlink structure and the uplink structure for the resource grid in LTE/IoT network are shown in Figure 7 and Figure 8 respectively. 

The smallest unit of the time-frequency resource of the LTE frame is the resource block, which consists of 12 grouped frequency subcarriers. The 10 ms frame consists of 10 subframes of 1 ms each (two slots of 0.5 ms each). The channel resource is allocated to the resource block (RB), where the 180 KHz bandwidth is transmitted to 12 subcarriers with a spread between the frequencies of 15 KHz. In the time domain, 7 OFDM symbols are transmitted in each slot (14 in the subframe). The channel distribution is shown Figure 7. 

When transmitting over the downlink at the physical level of the NB-IoT, the primary, secondary and NPSS (Narrow Band Synchronization Signals) synchronization channels are defined [61] as follows:

- Narrowband Physical Downlink Shared Channel (NPDSCH) data transmission.
- Narrowband Physical Downlink Control Channel (NPDCCH) control.
- Narrowband Physical Broadcast Channel (NPBCH) system information transmission.

Each frame starts with the transmission of the NPBCH channel, which may take zero subframe. Every 5th subframe is transmitted by NPSS signal, while the last subframe of each even numbered frame is transmitted by Narrowband Secondary Synchronization Signal (NSSS) signal. NPDSCH or NPDCCH channels are placed in the remaining free subframes. 

The base station in NB-IoT networks can operate with one or two antennas (R2000 and R2001 antenna ports). These ports transmit NB-IoT-specific reference signals. If the channel resource for NB-IoT is allocated in the bandwidth of the active LTE network, then the reference signals of the broadband network NRS1 and NRS2 are also transmitted to the Resource Block (RB). When placing the symbols of the NPDSCH channel, 1–3 OFDM symbols are reserved on the left side for transmission of the PDCCH control channel of the broadband LTE network (2 OFDM symbols in Figure 7). 

Here, we provide a modification for the control channels that consist of LTE PDCCH, LTE Cell Specific Reference Signal channels, and intelligent queue consistency control channels on an IoT controller communicating with an end IoT device. The control channel also transmits information to the controller—information about resources’ usage. These control channels are proposed for the flexibility of managing the QoS at the link layer, which transmits signaling information about the resource block for a specific IoT sensor message with its priority and unique device identifier. In contrast to the known solutions, these channels make it possible to allocate a single resource block to transmit a small message from an IoT sensor and provide it with a minimum delay of 0.5 ms within the frame. These delays are especially important for real-time tactical IoT data. The signal channels are shown in red in the green frame in Figure 7. 

The following discusses the narrowband physical uplink shared channel and narrowband physical uplink control channel shown in Figure 8:

- Narrowband Physical Uplink Shared Channel (NPUSCH) is a physical channel used for uplink data transmission by the IoT Device. It may also carry the uplink control information. This channel is the counterpart of PDSCH channel in uplink.
- Narrowband Physical Uplink Control Channel (NPUCCH) is the Physical Uplink Control Channel (PUCCH) that provides the various control signaling. These signaling are known as Scheduling request, Downlink data Acknowledgement (ACK)/Negative-acknowledgement (NACK) signaling, and Channel Quality Indicator (CQI) information.

NB-IoT devices can transmit the responsive Hybrid Automatic Repeat Request (HARQ) feedback over a narrowband physical uplink shared channel (NPUSCH) or over a narrowband physical uplink control channel (NPUCCH). We propose different options for defining the physical structures of the NPUCCH, NPUSCH, and user multiplexing on the uplink (UL). We also propose to use a new transfer request signal for communication with the IoT controller.

NB-IoT UEs (User Equipment) can transmit the responsive HARQ feedback over a narrowband physical uplink shared channel (NPUSCH) or a narrowband physical uplink control channel (NPUCCH). Options for defining the physical structures of the NPUCCH and NPUSCH and user multiplexing on the uplink (UL) are provided.

In the following section, we provide simulation results performed on the “Smart Queue” concept of the IoT controller.

## 6. Simulation Results of Developed Concept

**Figure 9:**
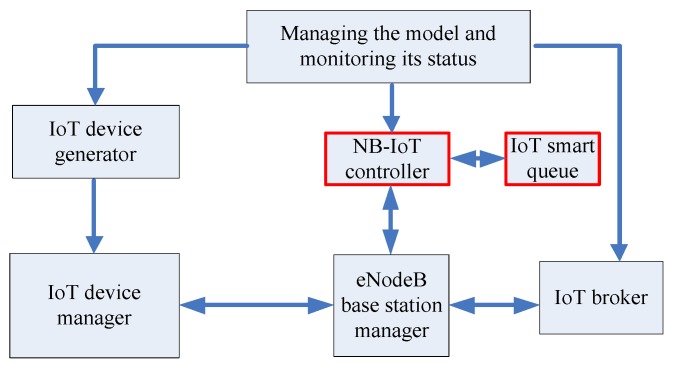
The simulation model for the LTE/IoT integrated solution.

To investigate the effectiveness of the proposed concept, we performed a set of simulations for the LTE/NB-IoT integrated solution. We implemented it in the form of a discrete events simulator and developed our java-based simulator for LTE/Nb-IoT. We also utilized the well-known tool named Discrete-Event Simulation and Modelling in Java DESMO-J (DESMO-J). It provides features such as queues, random number generation, and various statistical distributions. A simplified structural scheme of the simulation model is shown in Figure 9. 

The main elements of the simulation model that correspond to the real components of the network are as follows:

- The IoT device is a network endpoint with a QoS priority set under an SLA (Service-level agreement) contract according to Table.1. The main function of the IoT device is to generate a message, send a request for data transmission, send a message, receive a response message, and plan for the next data transfer procedure.
- The NB-IoT controller is responsible for monitoring the channel resources status at the base station. This includes message transmission, allocates necessary channel resources for specific IoT devices, the redistribution of channel resources between mobile and IoT devices, collection of data, processes and analyses of statistical data of successful and failed connections and transmissions.
- The eNodeB base station manager’s main function is to carry out message integrity verification, and provide interaction between IoT devices, the IoT controller, and the IoT broker. eNodeB contains an array of channel resources that the IoT-controller allocates for transmission for the IoT devices.
- The IoT broker stores data sent by IoT devices, analyzes it, and performs certain previously defined operations, such as transmitting, processing, and storing.

An IoT device generates an information message and sends the request for a channel resource allocation to the eNodeB base station. The request also contains the size of the message and the modulation type. At the base station, the integrity of the request is checked and then the message is redirected to the IoT controller. The IoT controller analyzes the request and the state of the channel resource array of the current base station. If the available channel resources are able to provide the required service within an allowable delay, then these resources are reserved for this IoT device. The IoT controller responds with the channel resource number to the current base station. The base station redirects the response to the IoT device, which analyzes the response and waits for its channel resource, in which it will transmit an information message through the base station to the IoT broker. At the same time, the IoT broker saves the information transmitted in the message for future use. If there are no free channel resources, then the request is served according to the above described algorithms.

During our performed simulation, we set forward predefined data. This includes the following:

- The number of IoT devices is set to 2000.
- The number of resource blocks within the narrowband of 200 KHz spectrum. The maximum number of resource blocks that can be transmitted in 1 s is 2000. In the simulation process, the number of resource blocks is a variable number which depends on the generated IoT message size and its requirement to the bandwidth.
- The modulation types are Binary Phase-Shift Keying (BPSK) and Quadrature Phase-Shift Keying (QPSK).
- The average length of a message from the IoT device depends on the modulation type used and is around 10 resource blocks.
- The average loads, *ρ_i_*, where i = 1,2,3,4,5 considered for an IoT controller are *ρ*_1_ = 0.12, *ρ*_2_ = 0.18, *ρ*_3_ = 0.5, *ρ*_4_ = 0.75, *ρ*_5_ = 1.
- The ratio of IoT devices per class are R_L1 = 10%, R_L2 = 20%, R_L3 = 30%, and R_L4 = 40%.
- The allowable delays for each class (Table 1) are D_L1 = 10 ms, D_L2 = 20 ms, D_L3 = T_3 Permissible_, D_L4 = T_4 No critical_.
- Types of delay are signal propagation speed over the wireless channel, packet processing time at base station, signal propagation time over wired channel, packet processing time at IoT controller, packet processing time by IoT device, and transmission awaiting time by IoT device.

A delay of the IoT data transmission end-to-end is calculated using Equation (1):

(1) $$T_{E2E_{delay}}=3\times t{\;}_{s.\;p.\;r.}+3\times t_{p.\;BS}+3\times {t.}_{s.\;p.\;c}+t_{p.\;IoTc.}+t_{p.\;IoT\;d.}+t_{w}$$

where, *t_s.p.r._ (t_signal propagation ratio)_* is a delay of a signal propagation over the wireless channel, *t_p.BS_ (t_processing BS_)* is the delay of a signal processing time at the base station, *t_s.p.c._ (t_signal propagation cable_)* is the delay of a signal propagation over a wired channel, *t_p.IoT.c._ (t_processing IoT controller_)* is the delay of the processing time at the IoT controller, t_processing IoT device_ is the delay of the processing time at the IoT device, and the delay caused by the IoT device awaiting transmission is *t_w_ (t_waiting for data transfer_)*. 

**Figure 10:**
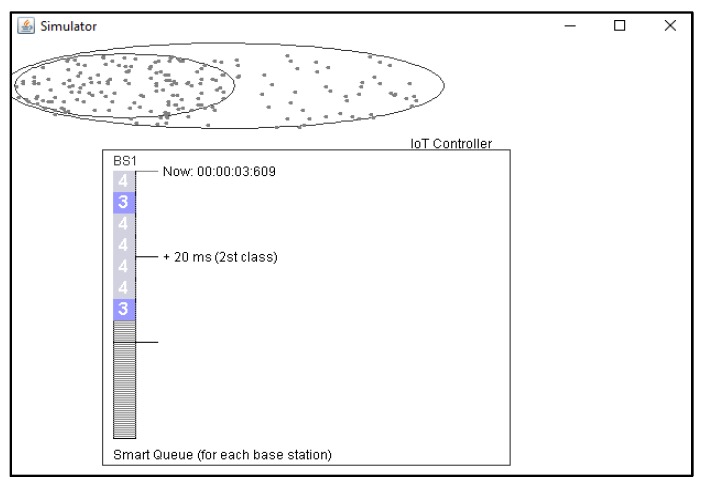
Simulation organization of the LTE/NB-IoT network.

The simulation model of the LTE/IoT integrated solution is depicted in Figure 10.

To demonstrate the efficiency of the proposed concept, we performed our simulation on various loads of the IoT controller. The investigation consists of two main simulation phases:

***Phase* I.** This phase focuses on E2E QoS when processing the incoming flow of the requests using the Proportional Fair Scheduling method described in works [62,63,64].

***Phase* II.** This phase focuses on E2E QoS when processing the incoming flow of requests using our proposed traffic prioritization *(P.IoT)* method.

During each simulation conducted, 100 frames of 10 ms duration are transmitted. Each frame contains 20 resource blocks of 0.5 ms duration. This means that 2000 resource blocks are transferred to one eNodeB per second. At every phase, we compare between the Prioritizing IoT traffic method and the Proportional Fair Scheduling method.

### 6.1. Scenario 1: An Average of 12% Load of IoT Controller 

**Figure 11:**
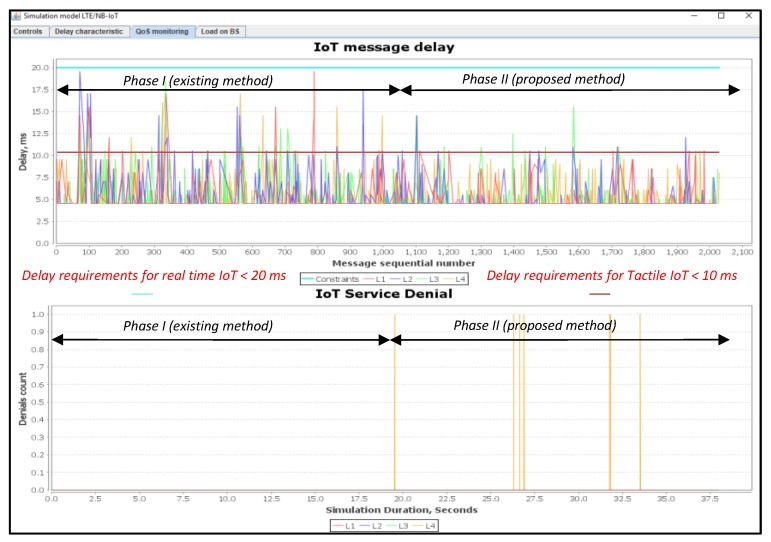
Delay and service denial values when serving requests from IoT devices (average load of IoT controller *ρ* = 12%).

In this simulation scenario, we used a 12% load. Figure 11 shows a comparison of IoT message delays and service denial value with different priorities. 

L1 and L2 represent real-time traffic, while L3 and L4 represent not real-time traffic. Thus, the proposed method is efficient for the real-time traffic. This method provides the minimum delay required for the tactile IoT messages. At 12% load, the Proportional Fair Scheduling method meets the requirements of the real-time traffic L2 (blue curve). For example, the delay of the transmission of L2 class should not exceed D_L2 = 20 ms. For L1 (tactical IoT), it should not exceed D_L1 = 10 ms. 

It should be noted that for some messages of class L1 (red curve) in the Proportional Fair Scheduling, the delay exceeds the requirements of D_L1 = 10 ms. Loss of message transmission requests for classes L1 to L4 are absent. This is because the system works at low load. When using our proposed method, the delay for both L1 and L2 in real-time classes is within the desired or setup requirements. However, there are some losses of requests for non-real-time traffic of L4 class shown in Figure 11, and this is clearly described in our proposed algorithm. L4 message losses are forced according to our proposed algorithm developed and described in Figure 5. The occurrence of L4 query losses is due to the need to release some resources for L1 and L2 priority messages. Thus, by releasing the required resources for the L1 and L2 service classes, the data transmission delay does not exceed the allowable limits.

**Figure 12:**
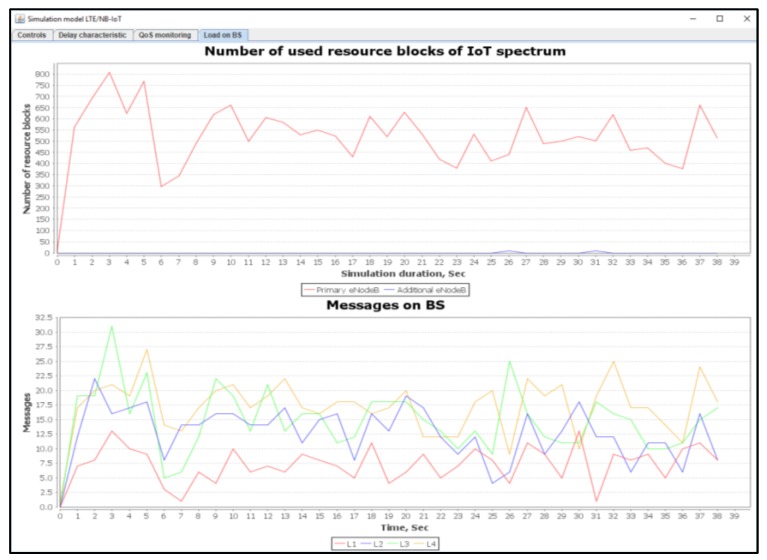
Used IoT spectrum resource blocks and transmitted messages ratios with different priorities (average load of IoT controller *ρ* = 12%).

Figure 12 shows a number of used resource blocks of IoT spectrum and the ratio of the transmitted messages of different priorities.

**Figure 13:**
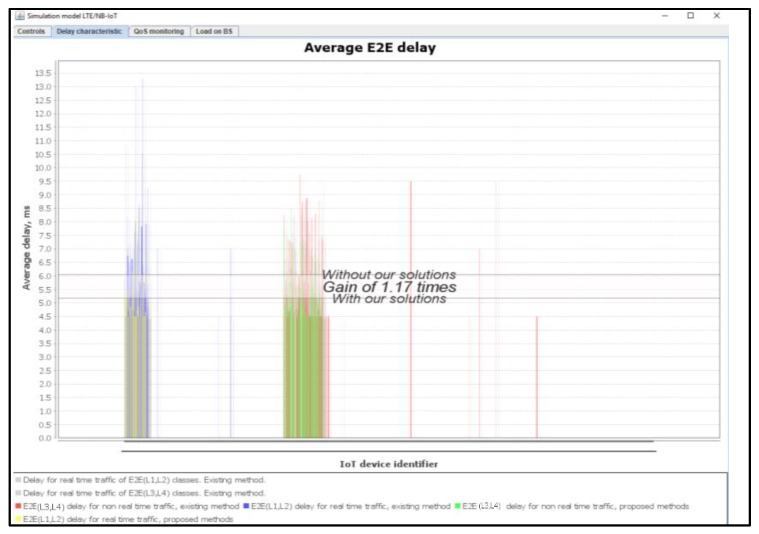
Estimated average E2E delay gain of IoT services with our solution (average load of IoT controller *ρ* = 12%).

Figure 13 shows the average delays of IoT messages of the classes L1, L2, L3, and L4. We claim that the use of our proposed method provides a gain of 1.17 times as compared to the Proportional Fair Scheduling method. Additionally, the modeling results showed the average delays of IoT messages did not exceed the defined requirements of D_L1 = 10 ms and D_L2 = 20 ms.

### 6.2. Scenario 2: An Average of 18% Load of NB-IoT Controller

**Figure 14:**
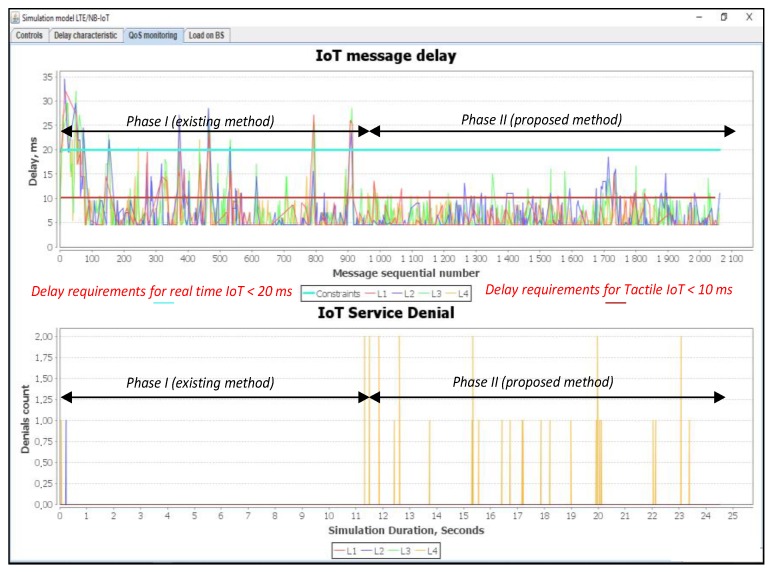
Delay and service denials for the requests from IoT devices (average load of IoT controller *ρ* = 18%).

In this simulation scenario, we used an 18% load. As can be seen from the simulation results presented in Figure 14, the Proportional Fair Scheduling method does not provide the necessary delay requirements for both L1 and L2 real-time traffic, i.e., messages transmission delay exceeds D_L1 = 10 and D_L2 = 20 ms. 

**Figure 15:**
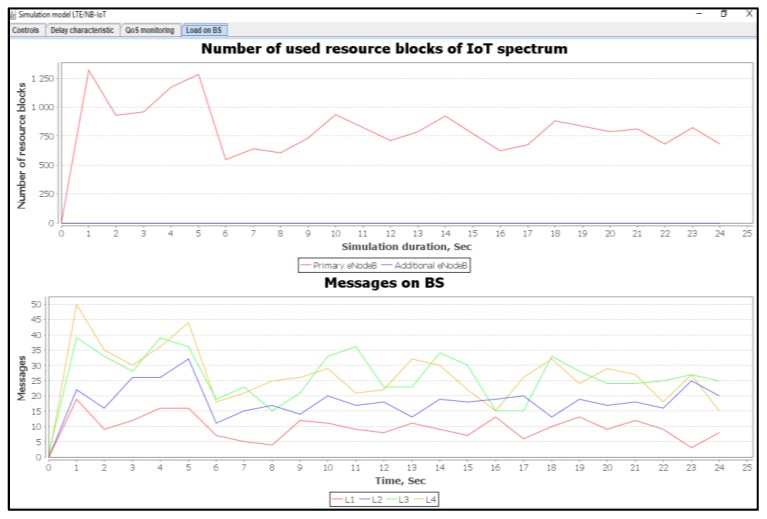
Number of IoT spectrum resource blocks and transmitted messages ratio with different priorities (average load of IoT controller *ρ* = 18%).

When using our proposed method, the delays for the real time class L2 are within the predefined requirements. For the L1 class, only 0.1% of all transmitted messages was subject to a higher delay that ranges from 1 to 5 ms. According to our proposed algorithms described in Figure 2 and Figure 4, there are losses for non-real-time traffic of the L3 class. Thus, by releasing the required resources for the L1 and L2 service classes, the data transmission delay does not exceed the allowable limits. Figure 15 shows the number of used resource blocks of the IoT spectrum and the transmitted messages ratio with different priorities.

**Figure 16:**
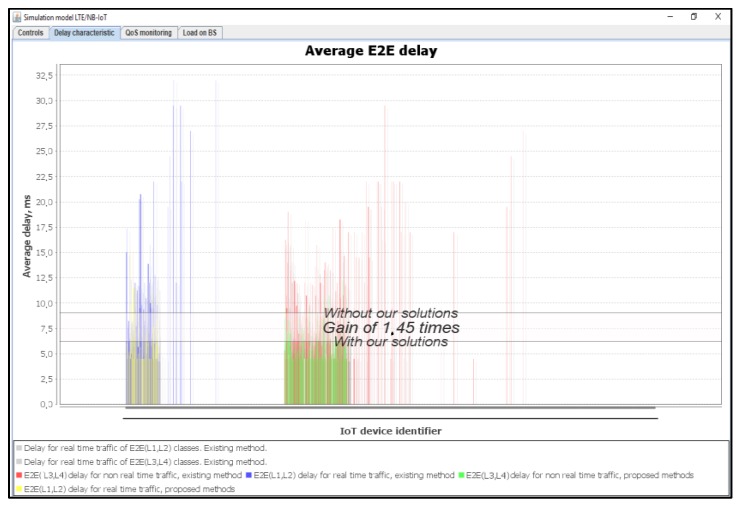
Average E2E delay gain for IoT services—our solution (average load of IoT controller *ρ* = 18%).

Figure 16 shows the average delays of IoT messages for classes L1, L2, L3, and L4. We observed that our proposed method provides a gain of 1.45 times better than the Proportional Fair Scheduling method. Additionally, the modeling results showed that the average delays of IoT messages did not exceed the required time delay of D_L1 = 10 ms and D_L2 = 20 ms.

### 6.3. Scenario 3: An Average of 50% Load of IoT Controller

**Figure 17:**
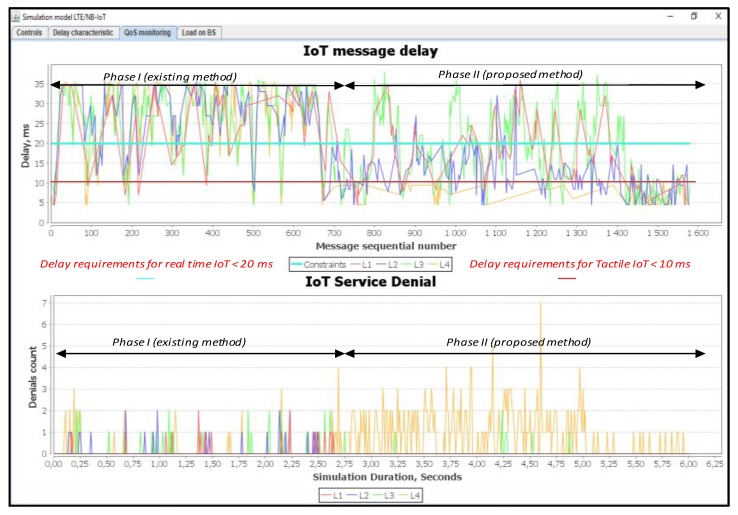
Delay and service denials for service requests from IoT devices (average load of IoT controller *ρ* = 50%).

When we used a 50% load, the Proportional Fair Scheduling method did not provide the necessary delay requirements for both class L1 and class L2 real-time traffic. The losses of class messages of L1 are equal to R_L2 = 25%, and of L2 are equal to R_L2 = 12%, as shown in Figure 17. With our method, the delays for the real time class L1 are within the predefined requirements. For class L2, it is only 0.5% of all transmitted messages where they were subject to a high delay. This is clearly way above the norm, which varies from 1 to 14 ms. The losses R_L1 of class L1and R_L2 of class L2 messages are 0% using our proposed method. With reference to our proposed algorithms, there were some loss of messages’ requests for the traffic of non-real-time of class L3 and class L4. These messages’ losses happened due to the release of the required resources for class L1 and class L2 services to provide the required/allowable delays.

**Figure 18:**
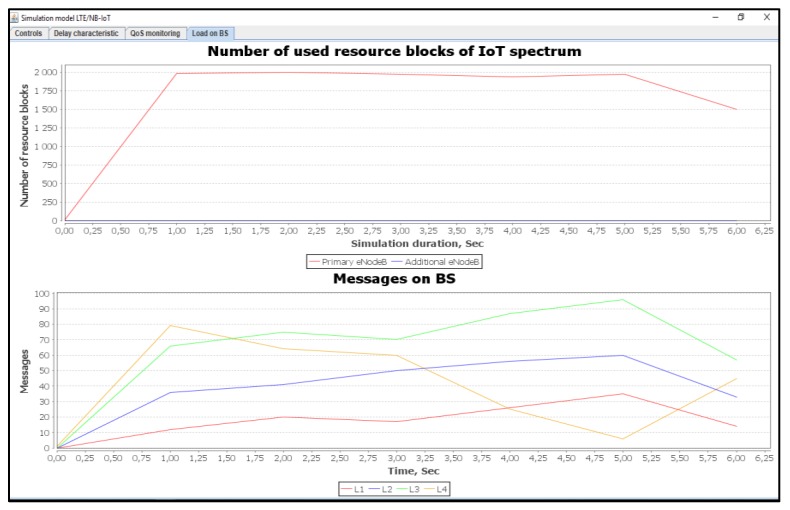
Used IoT spectrum resource blocks and transmitted messages ratio with different priorities (average load of IoT controller *ρ* = 50%).

Figure 18 shows the number of used resource blocks of IoT spectrum. It also shows the transmitted messages ratio with different priorities. 

**Figure 19:**
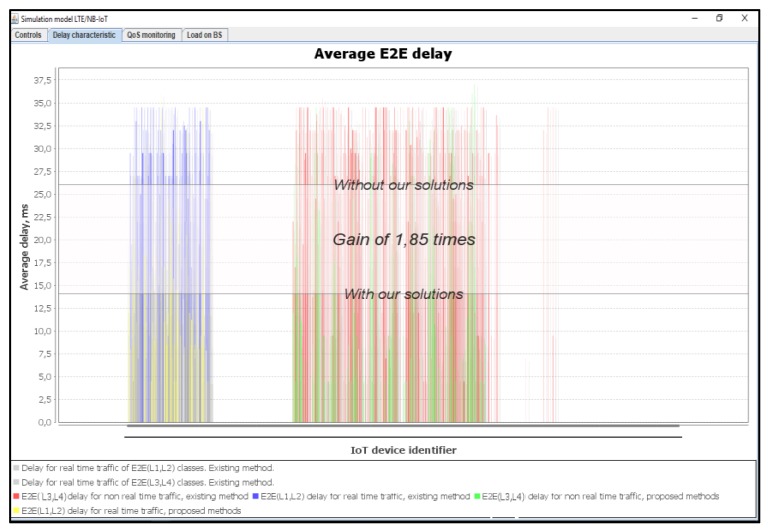
Average E2E delay gain of IoT services with our solution (average load of IoT controller *ρ* = 50%).

Figure 19 shows the average delays of IoT messages of classes L1, L2, L3, and L4. It is demonstrated that the use of our proposed method gives a gain of 1.85 times better than the Proportional Fair Scheduling method. Furthermore, the modeling results showed that the average delays of IoT messages with the Proportional Fair Scheduling method exceeded the expected delay requirements of D_L2 = 20 ms, while our proposed method did not exceed D_L2 = 20 ms for real-time traffic.

### 6.4. Scenario 4: An Average of 75% Load of IoT Controller

**Figure 20:**
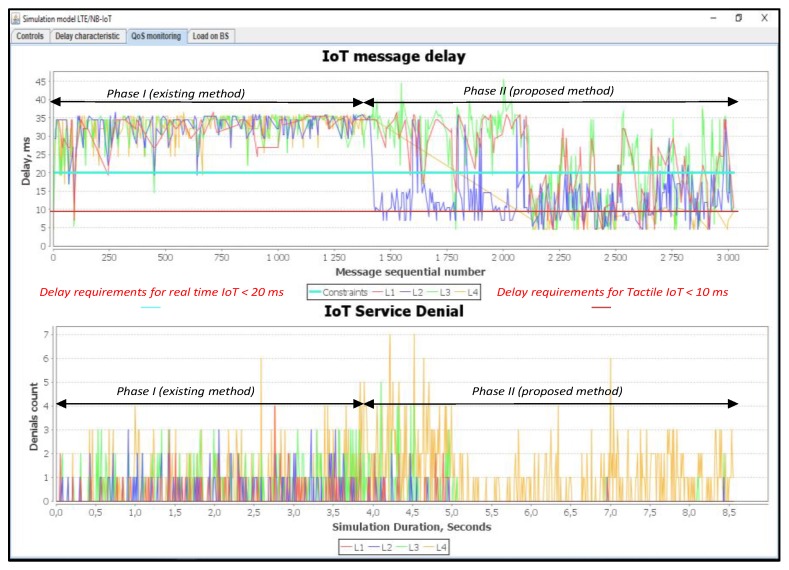
Delay and service denials from IoT devices (average load of IoT controller *ρ* = 75%).

In this simulation scenario, we used a 75% load. In this situation, the Proportional Fair Scheduling method did not meet the necessary delay requirements for both real-time traffic class L1 and class L2. The losses of messages of class L1 are equal to R_L1 = 55%, while the losses for class L2 are equal to R_L2 = 65%, as shown in Figure 20. Using our proposed method, the losses of class L1 are equal to R_L1 = 1% and the same percentage is achieved with class L2.

**Figure 21:**
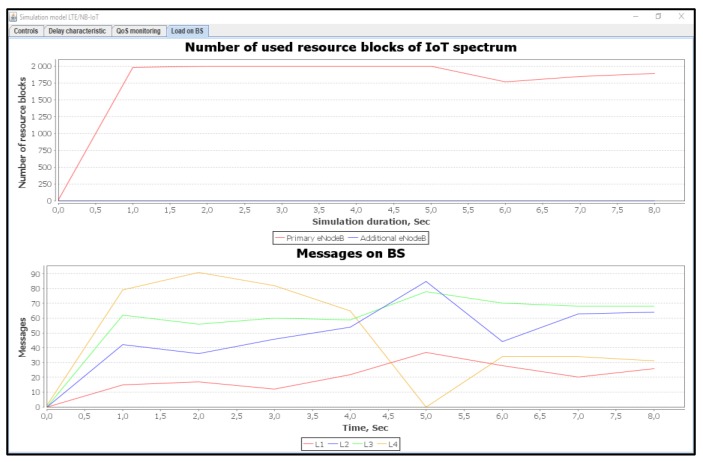
Used IoT spectrum resource blocks and transmitted messages ratio for different priorities (average load of IoT controller *ρ* = 75%).

Figure 21 shows the number of used resource blocks of IoT spectrum. This figure also shows the transmitted messages ratio with different priorities. Moreover, the average delays of IoT messages of class L1, L2, L3, and L4 are also shown. 

**Figure 22:**
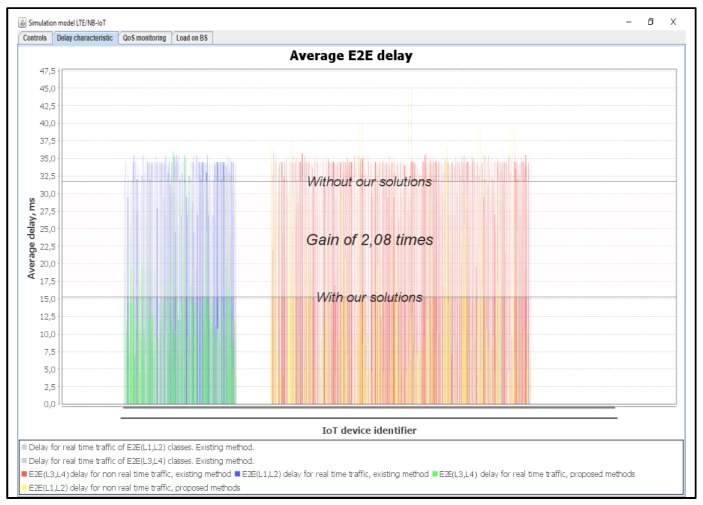
Average E2E delay gain of IoT services for our solution (average load of IoT controller *ρ* = 75%).

Our method provides a gain of 2.08 times better than the Proportional Fair Scheduling method, as shown in Figure 22. The results showed that the average delays of IoT messages with the Proportional Fair Scheduling method exceeded the allowed delay of 20 ms, with an average delay of 32 ms. With our method, the average delay achieved is 15.38 ms.

### 6.5. Scenario 5: An Average of 100% Load of IoT Controller

**Figure 23:**
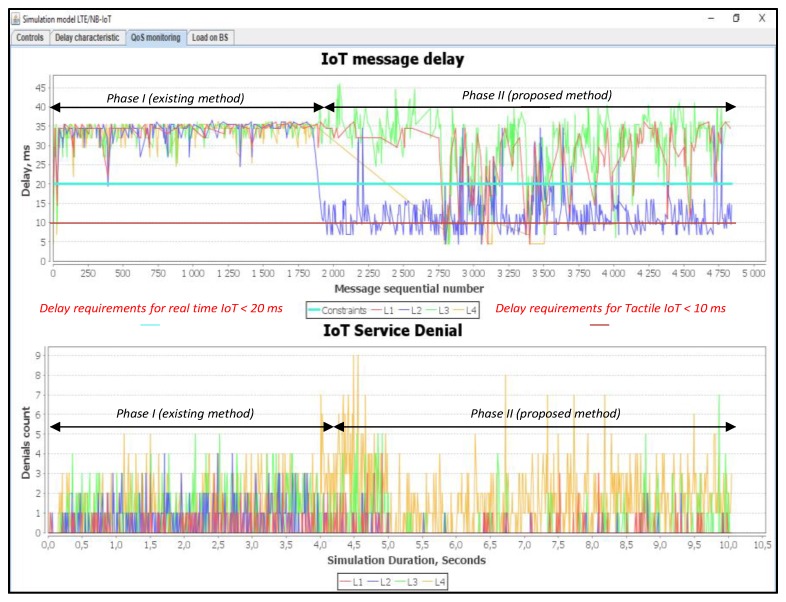
Current delay and service denials count when servicing requests from IoT devices (average load of IoT controller *ρ* = 100%).

In this simulation scenario, we used a 100% load. With using the Proportional Fair Scheduling method, the results of messages loss R_L1 in class L1 is equal to 64% and in class L2 the loss R_L2 is equal to 73%, as shown in Figure 23. In contrary to our proposed method, the loss of requests R_L3 and R_L4 for non-real-time traffic of classes L3, L4 are equal to 26%, whereas, losses R_L2 and R_L1 in class L1 and L2 are equal to 4%, as shown in Figure 23. 

**Figure 24:**
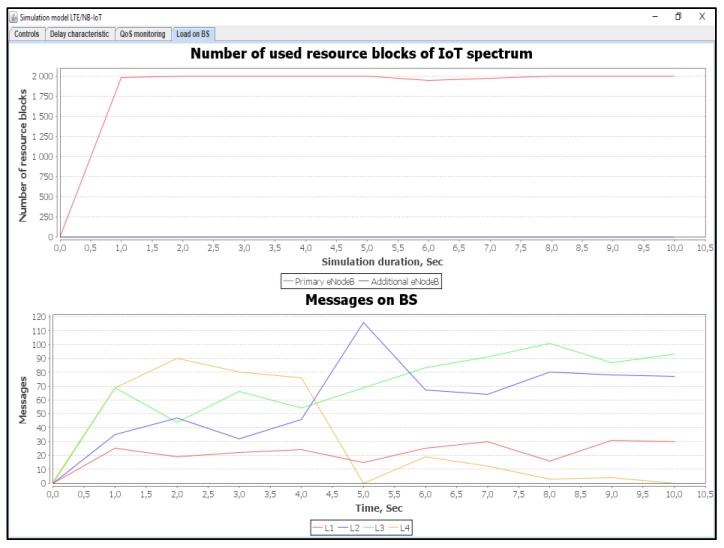
Used IoT spectrum resource blocks and transmitted messages ratio with different priorities (average load of IoT controller *ρ* = 100%).

Figure 24 shows the number of used resource blocks of IoT spectrum. This figure also shows the transmitted messages ratio for different priorities. 

**Figure 25:**
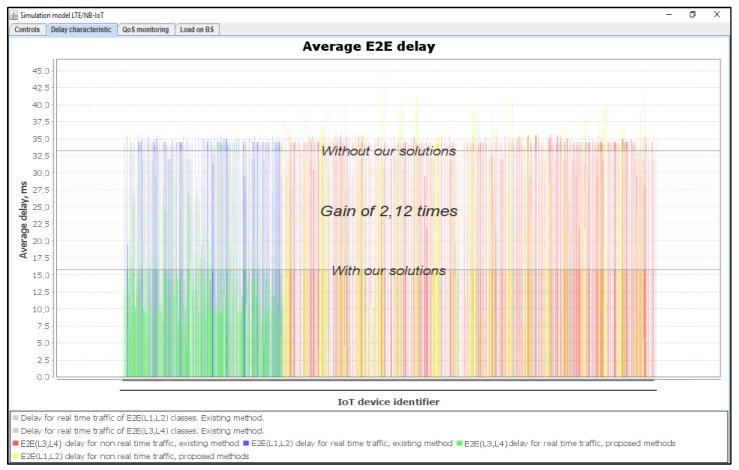
Average E2E delay gain of IoT services with our solution (average load of IoT controller *ρ* = 100%).

**Figure 26:**
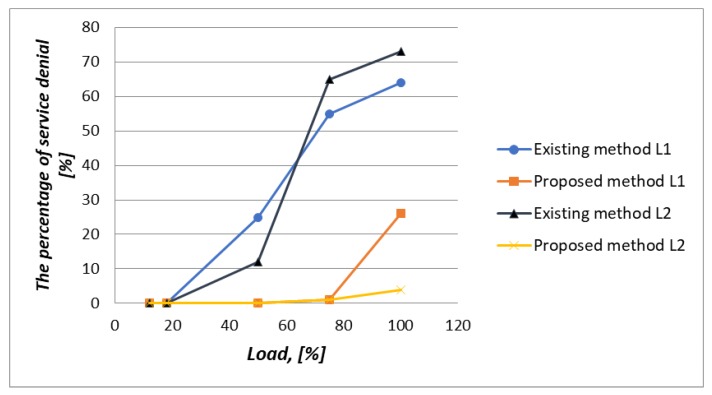
Service denials for priority devices (IoT devices of classes L1 and L2) with existing and proposed methods.

Figure 25 shows the average delays of IoT messages of class L1, L2, L3, and L4. Again, our proposed method in comparison with the Proportional Fair Scheduling method gives a gain of 2.12 times. It is clear that the average delays of IoT messages using the Proportional Fair Scheduling method exceeds the maximum allowed delay of D_L2 = 20 ms, with an average of D_L2 = 33.5 ms. However, using our proposed method, the average delay is only D_L2 = 15.8 ms. Service denials for priority devices (IoT devices of classes L1 and L2) with existing and proposed methods is depicted in Figure 26.

**Figure 27:**
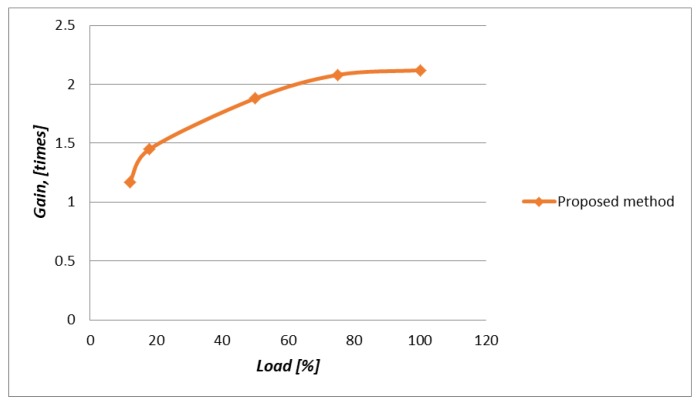
Efficiency of the proposed method at different loads.

From the results of the simulation, we observe that the method of IoT traffic prioritization provided a reduction in the average E2E delay for devices transmitting data in real-time (L1, L2). This is due to an increase in the average transmission delay for devices that are not susceptible to delay (L3, L4). The efficiency of the proposed method at different loads is depicted in Figure 27.

The prioritization method allowed the reduction in the number of service denials by 38% for class L1, and 69% for class L2, when it is compared with the Proportional Fair Scheduling method under high load conditions.

## 7. Discussion

Unlike the standard known NB-IoT architecture, our proposed NB-IoT architecture reduces the overload from the base station when planning radio resources. This is due to the fact that the existing LTE/NB-IoT architecture has a controller that acts as a resource scheduler for NB-IoT devices located in the eNodB LTE base station, which also includes a resource scheduler that is responsible for the allocation and planning of mobile subscriber resources. Scheduling is a process through which eNodeB decides which UEs (user equipment) should be given resource blocks (RBs), and how much resource (RBs) should be given to send or receive data. In classical NB-IoT structure, scheduling is done at per subframe basis every 10 millisecond. The entity which is governing this is known as the scheduler. Such a joint combination of controllers leads to a significant load of eNodeB due to the simultaneous connection of a large number of IoT devices, namely, it leads to an increase in CPU and RAM of the base station and as a result of the degradation of QoS parameters. Exchanging for new base stations with better computing characteristics is an expensive way, and mass deployment of the NB-IoT will require a significant number of replacement LTE base stations. To avoid this problem, we suggest separating the NB-IoT controller on a separate server, which will be responsible only for the maintenance and resource planning for IoT devices. With the significant increase in load from IoT devices, a situation may occur that will lead to a significant increase in service delay, which is not desirable for critical real-time IoT services. Our proposed solution will allow easy replacement of the server with another server with better computing characteristics, and will reduce the processing time by the controller, which directly affects the E2E delay.

In our work, we developed the architecture of the integrated mobile access network LTE/NB-IoT for 4G and 5G networks. We modified the structure of NB-IoT frame, where a logical data channel is allocated to reduce the delay and communication of the NB-IoT controller. Unlike known solutions, these channels allow the allocation of one resource block to transmit a small message from the sensor IoT and provide a minimum delay of 0.5 ms in the frame. The signal channels are shown in red in the green frame in Figure 10 and Figure 11. The use of the existing NB-IoT technology is niche and used for non-delay-critical internet applications due to the quality of service limitations. If you need to implement LTE -based 4G/5G access networks with critical requirements, it will be possible to use the concept of NB-IoT technology that we offer in this paper.

Based on the results of the simulation, we found that the proposed NB-IoT system effectively provides the acceptable value of the delay and denial count for each real time IoT service under loads of the IoT controller from 0%–70%. Under high load conditions (70%–100%), the system performs better than the existing method, but for not all IoT critical services are the acceptable value of the delay and denial count ensured, in particular, the results of which are shown in Figure 26 at a 100% load of the IoT controller. That is why in the future, we plan to develop a new method to ensure that all IoT messages are delivered in real-time with the necessary QoS parameters under high-load conditions in our proofs of concepts NB-IoT technology.

## 8. Conclusions

Compared to all previous generations, LTE networks achieve lower delays in data transmission due to fewer intermediate elements. Thus, the use of LTE networks to interact with IoT elements should bring additional revenues to operators and give them an impulse to further investment growth.

This paper described a proposed solution for the Tactile IoT to transfer data with ultra-short delays, based on NB-IoT technology. We discussed our performed modifications to the LTE architecture by transferring part of the functions from the base station, eNodeB, to the NB-IoT controller. This controller performed the downlink and uplink channel planning for the IoT devices, which allowed the network operators to keep existing base stations eNodeB unchanged. We modified the structure of NB-IoT frame, where a logical data channel is allocated to reduce the delay and communication of the NB-IoT controller. The proposed solution extends the functionality of IoT control in real time for LTE-based 4G/5G networks.

Further in this paper, we proposed a prioritization method within the IoT traffic to provide E2E QoS in the integrated LTE/NB-IoT network. We developed an algorithm for managing a “smart queue” based on the IoT traffic prioritization procedures. 

With the number of simulations, we demonstrated that our proposed approach ensured high end-to-end QoS of the real-time traffic. This was achieved by reducing an average end-to-end transmission delay of the real-time messages from 1.17 to 2.12 times as compare to the Proportional Fair Scheduling method. 

## Figures and Tables

**Table 1:** Characteristics of Quality of Service Class Identifier IoT (QCI_IoT_)

QCI_IoT_	Type	Priority	Allowed Delay D, ms	Allowed Service Denial Count, R%	IoT Service Class
1	Guaranteed transmission delay for real time traffic (GBR_IoT_)	1	10	0.01	L1
2		2	20	0.1	L2
3	Guaranteed transmission delay for non-real time traffic (GBR_IoT_)	3	1000	5	L3
4	Non-guaranteed transmission delay for non-real time traffic (Non-GBR_IoT_)	4	undefined	undefined	L4
